# FGsub: *Fusarium graminearum* protein subcellular localizations predicted from primary structures

**DOI:** 10.1186/1752-0509-4-S2-S12

**Published:** 2010-09-13

**Authors:** Chenglei Sun, Xing-Ming Zhao, Weihua Tang, Luonan Chen

**Affiliations:** 1 Institute of Systems Biology, Shanghai University, Shanghai 200444, China; 2 School of Communication and Information Engineering, Shanghai University, Shanghai 200444, China; 3 National Key Laboratory of Plant Molecular Genetics, Institute of Plant Physiology and Ecology, Shanghai Institutes for Biological Sciences, Chinese Academy of Sciences, 300 Fenglin Road, Shanghai 200032, China; 4 Key Laboratory of Systems Biology, SIBS-Novo Nordisk Translational Research Centre for PreDiabetes, Shanghai Institutes for Biological Sciences, Chinese Academy of Sciences, China

## Abstract

**Background:**

The fungal pathogen *Fusarium graminearum* (telomorph *Gibberella zeae*) is the causal agent of several destructive crop diseases, where a set of genes usually work in concert to cause diseases to crops. To function appropriately, the *F. graminearum* proteins inside one cell should be assigned to different compartments, i.e. subcellular localizations. Therefore, the subcellular localizations of *F. graminearum* proteins can provide insights into protein functions and pathogenic mechanisms of this destructive pathogen fungus. Unfortunately, there are no subcellular localization information for *F. graminearum* proteins available now. Computational approaches provide an alternative way to predicting *F. graminearum* protein subcellular localizations due to the expensive and time-consuming biological experiments in lab.

**Results:**

In this paper, we developed a novel predictor, namely FGsub, to predict *F. graminearum* protein subcellular localizations from the primary structures. First, a non-redundant fungi data set with subcellular localization annotation is collected from UniProtKB database and used as training set, where the subcellular locations are classified into 10 groups. Subsequently, Support Vector Machine (SVM) is trained on the training set and used to predict *F. graminearum* protein subcellular localizations for those proteins that do not have significant sequence similarity to those in training set. The performance of SVMs on training set with 10-fold cross-validation demonstrates the efficiency and effectiveness of the proposed method. In addition, for *F. graminearum* proteins that have significant sequence similarity to those in training set, BLAST is utilized to transfer annotations of homologous proteins to uncharacterized *F. graminearum* proteins so that the *F. graminearum* proteins are annotated more comprehensively.

**Conclusions:**

In this work, we present FGsub to predict *F. graminearum* protein subcellular localizations in a comprehensive manner. We make four fold contributions to this filed. First, we present a new algorithm to cope with imbalance problem that arises in protein subcellular localization prediction, which can solve imbalance problem and avoid false positive results. Second, we design an ensemble classifier which employs feature selection to further improve prediction accuracy. Third, we use BLAST to complement machine learning based methods, which enlarges our prediction coverage. Last and most important, we predict the subcellular localizations of 12786 *F. graminearum* proteins, which provide insights into protein functions and pathogenic mechanisms of this destructive pathogen fungus.

## Background

The fungal pathogen *Fusarium graminearum* (telomorph *Gibberella zeae*) is the causal agent of several destructive crop diseases. For example, *F. graminearum* causes destructive Fusarium head blight (FHB) on wheat and barley, and is a leading cause of economical loss for these crops [1]. It is estimated that *F. graminearum* causes economical losses of $3 billion in the United States of America between 1991 and 1996 [2]. In addition, the fungus contaminates grain with toxic metabolites that threat human health [3].

Therefore, it is necessary to investigate the mechanism underlying the pathogenic process of this destructive fungus, which can help to find an efficient way to control it. The annotation of *F. graminearum* proteins can provide insights into biological processes in which proteins are involved, and give hints to pathogen-host interactions. Generally, proteins are transported to specific compartments in a cell so that they can function properly. That is, the subcellular localizations can provide insights into protein functions and help understand the pathogenic process of this destructive fungus. Although the whole genome of *F. graminearum* has been sequenced and partly annotated [4], there are no subcellular localization annotation available for *F. graminearum* right now. Since it is expensive and time-consuming to determine protein subcellular localizations by biological experiments in lab, computational approaches provide alternative ways to predicting *F. graminearum* protein subcellular localizations.

In literature, a number of machine learning methods have bee developed for protein subcellular localization prediction, such as *k*-nearest neighbor classifiers [5-7], artificial neural networks [8-10], support vector machines (SVMs) [11-13], Bayesian networks [14-16], and so on. Furthermore, many different types of features, i.e. description of proteins, have been used for subcellular localization prediction. One popular description of protein is amino acid composition [17-20] including single amino acid composition (AA), pair amino acid composition (PAA) and gapped amino acid composition (GapAA). A number of works have used amino acid composition as the features of a protein owing to its simplicity and effectiveness [21-23].

For example, SubLoc [24] utilized support vector machine (SVM) and amino acid composition features to obtain high prediction accuracy. Nakashima and Nishikawa [22] used PAA, and Chou [11] used pseudo amino acid composition (PseAA) for prediction. PSORT [25] used various sequence features to predict protein localization sites in eukaryotic cells. TargetP [10] utilized artificial neural networks and N-terminal sequence to predict subcellular localizations. Except for sequence data, other information have also been found useful for subcellular localization prediction. Cai and Chou [26] integrated different information, including pseudo-amino acid composition, gene ontology information, and domain composition, for subcellular localization prediction. BaCelLo [27] used N-terminal, C-terminal, amino acid composition, and sequence alignment profile to predict subcellular localizations. Tamura et al [20] used alignment of block sequences to improve prediction accuracy. Despite the success of different methods, it is difficult to say which is better than another one. Li et al [28] presented a meta-predictor by combining strengths of multiple available predicting programs, and high prediction accuracy is therefore expected. Most recently, Hsu et al [13] developed a probabilistic latent semantic analysis method for Gram-negative bacteria. Ideker et al [7] integrated various information, including protein interaction network, gene ontology, hydrophobicity, side-chain mass and domain composition, and improved prediction accuracy significantly.

In all methods described above, subcellular localization prediction is actually formulated as a classification problem, where the proteins belonging to the target compartment are usually treated as positive samples while the rest are negative samples. Therefore, the number of negative samples is generally much larger than that of positive samples, which leads to imbalance problem and degrade the performance of the classifier [29]. Under the circumstances, we present a new algorithm to cope with imbalance problem arising from protein subcellular localization prediction. Furthermore, there are a large number of features extracted from protein sequence for each protein in the learning procedure, which leads to ‘bottleneck of dimensionality', and the noise in the data will degrade the performance of the classifier. In this work, feature selection techniques are utilized to find out most informative features for each subcellular localization. In addition, different features can make different contributions to protein subcellular localization prediction. Therefore, a novel ensemble classifier is developed to combine contributions from different features and thereby improves the prediction accuracy. Based on the methods described above, we developed a novel predictor, namely FGsub, to predict *F. graminearum* protein subcellular localizations from the primary structures, i.e. protein sequences. First, a non-redundant fungi data set with subcellular localization annotation is collected from UniProtKB [30] database and used as training set, where the subcellular locations are classified into 10 groups. Subsequently, Support Vector Machine (SVM) is trained on the training set and used to predict *F. graminearum* protein subcellular localizations for those proteins that do not have significant sequence similarity to those in training set. The performance of SVMs on training set with 10-fold cross-validation demonstrates the efficiency and effectiveness of the proposed method. In addition, for *F. graminearum* proteins that have significant sequence similarity to those in training set, BLAST is utilized to transfer annotations of homologous proteins to uncharacterized *F. graminearum* proteins so that the *F. graminearum* proteins are annotated more comprehensively.

Consequently, we predict the subcellular locations for 12786 *F. graminearum* proteins, which can provide insights into protein functions and pathogenic mechanisms of this destructive pathogen fungus. The data sets used in this work and the prediction results are available at http://csb.shu.edu.cn/fgsub/.

## Results and discussion

### Construction of balanced ensemble classifier

To predict subcellular localizations of *F. graminearum* proteins, a set of fungi proteins with subcellular localization annotations were extracted from UniprotKB and used as training set in this work. As shown in Table 1, the non-redundant data set contains 4592 proteins belonging to 10 subcellular locations, and the details of processing data can be found in Methods. The classifier used here are Support Vector Machines (SVMs), which was implemented with LIBSVM [31]. The gaussian kernel was employed in SVMs at all stages of the classification procedure, and the parameters were optimized with 10-fold cross-validation. In the training of SVM classifiers, we adopted the one-versus-rest method, where proteins belonging to the corresponding subcellular localization were regarded as positive set while others as negative set. For each of the 10 subcellular locations, one ensemble classfier consists of a series of SVM classifiers was trained and a protein was predicted to be positive or negative by corresponding classifier.

**Table 1:** Distributions of the fungi proteins with known subcellular localizations, where only localizations with more than 30 annotations are shown for clarity.

Localization	Proteins in UniProtKB^*a*^	Proteins_40^*b*^
Extracellular	272	148
Cytoplasm	1357	916
ER	895	561
Golgi apparatus	276	150
Nucleus	1538	1354
Mitochondrion	1719	949
Peroxisome	120	82
Endosome	105	54
Vacuole	315	192
Cell membrane	351	186
Total	6948	4592

*a* Number of proteins with unique localization found in UniProtKB.

*b* Curated data set with pairwise sequence identity <40%.

To evaluate the performance of our method, AUC (area under ROC curve) score was employed in this work. First, different features were evaluated for selecting the most informative features. Table 2 lists the 10-fold cross-validation results by SVM classifiers based on different features for different compartments, where threAA means feature description based on three amino acids, N-term and C-term respectively means feature description based on N-terminal and C-terminal sequence, and GapAA means features extracted based on gapped amino acids but with different gaps. For the gapAA information, only two best representation are shown in the table, i.e Gap1 and Gap2. In addition, the optimal combination of different classifiers trained on different features was found in 10-fold cross-validation. In Table 2, the bold elements without strikethrough denote the corresponding classifiers were chosen to be integrated for the final ensemble classifier and vice versa. From the results, we can see that different features make different contributions to distinct compartments, and the classifier with low prediction accuracy may also make contribution to the ensemble classifier due to the diversity introduced by it. Meanwhile, we can see that threAA, N-term, and C-term are the features that contribute most to the predictions. For threAA, each threAA can be treated as a sequence motif, which determines protein function. N-term and C-term have relation to signal peptides, which play important roles in protein subcellular localizations. Therefore, it is not surprise to find that these three features contribute most to predictions. It is also found that the ensemble classifier that fuse results by single classifiers indeed improve prediction accuracy.

**Table 2:** The 10-fold cross-validation results by SVM classifiers based on different features and those by ensemble classifiers for 10 locations with respect to AUC scores, where the ensemble classifiers are the optimal combinations of different SVM classifiers trained on features without strikethrough. The numbers with strikethrough indicate the corresponding classifier was not used in the ensemble classifier. The numbers within the brackets denote the corresponding Gap.

Localization	threAA	N-term	C-term	Gap1	Gap2	Ensemble
Extracellular	**0.909**	**0.891**	0.812	**0.943(13)**	0.943(7)	0.950
Cytoplasm	****0.652****	****0.653****	****0.643****	****0.637(15)****	****0.630(5)****	0.738
ER	**0.789**	**0.681**	**0.703**	**0.811(11)**	**0.806(13)**	0.827
Golgi apparatus	**0.748**	**0.791**	**0.764**	**0.732(7)**	0.729(5)	0.848
Nucleus	**0.688**	**0.630**	**0.615**	**0.709(13)**	**0.707(15)**	0.721
Mitochondrion	**0.722**	**0.781**	0.665	**0.805(15)**	0.802(13)	0.833
Peroxisome	**0.808**	**0.797**	**0.777**	**0.816(15)**	**0.815(13)**	0.882
Endosome	**0.705**	**0.787**	**0.795**	**0.860(13)**	**0.848(11)**	0.895
Vacuole	**0.730**	**0.749**	**0.758**	**0.748(15)**	0.746(9)	0.820
Cell membrane	**0.798**	**0.792**	**0.786**	**0.801(11)**	**0.800(9)**	0.837

**Figure 1:**
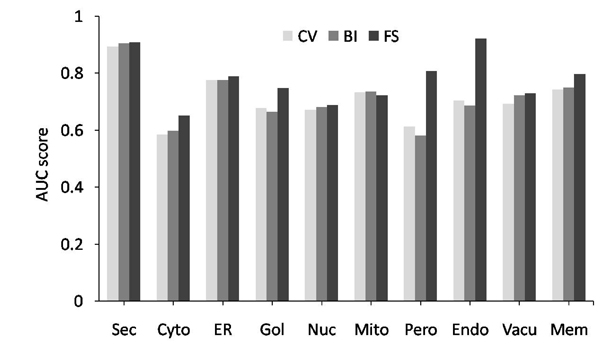
The comparison of performance of SVM classifiers without feature selection against those with feature selection and balancing, where the results were obtained with 10-fold cross-validation.

Next, we investigated the effects of balancing and feature selection on performance of classifier. In this work, the feature threAA was taken as an example. Figure 1 shows the comparison of performance of SVM classifiers without feature selection against those with feature selection and balancing, where CV in figure 1 means the 10-fold cross-validation results without feature selection and balancing, while BI means the results with balancing, and FS means the results with feature selection and balancing. From the results, we can see that feature selection and ensemble classifier can really significantly improve prediction accuracy, which demonstrates the efficiency and effectiveness of the proposed method.

### Comparison with other methods

Since there are a number of methods have been developed for protein subcellular localization prediction, to see the performance of our method, we compared our method with several existing methods. In this part, our method was compared with PLOC [12] and PSLDoc [13]. PLOC uses the compositions of amino acids and amino acid pairs, and PSLDoc uses the gapped-dipeptides *XdZ* with gap *d* (0 ≤ *d* ≤ 13) between any two amino acids *X* and *Z*. For fair comparison, SVM classifiers were used for all methods. Table 3 shows the comparison of prediction accuracies by different methods on the fungi data sets based only on protein sequence information, where the results were obtained with 10-fold cross-validation. From the results, we can see that our method outperforms PLOC and PSLDoc in almost all 10 subcellular localizations except Nucleus. Especially, the AUCs for ‘Golgi apparatus', ‘Peroxisome' and  ‘Endosome' are higher than those of the other two methods with a magnitude about 0.1. The comparison with other methods based on the same data set demonstrate that our proposed balanced ensemble classifier is really effective and efficient for predicting fungi protein subcellular localizations. Note that the aim of this work is not to compare different methods instead the work tries to predict protein subcellular localizations for *F. graminearum*.

**Table 3:** Comparison of FGsub with PLOC and PSLDoc based on the 10 fold cross-validation on the fungi data set with respect to AUC scores.

Localization	PLOC	PSLDoc	FGsub
Extracellular	0.9220	0.9140	**0.950**
Cytoplasm	0.6572	0.6668	**0.738**
ER	0.7813	0.8083	**0.827**
Golgi apparatus	0.7314	0.7090	**0.848**
Nucleus	0.7088	**0.7331**	0.721
Mitochondrion	0.7972	0.8069	**0.833**
Peroxisome	0.6335	0.6684	**0.882**
Endosome	0.8031	0.7993	**0.895**
Vacuole	0.7141	0.7476	**0.820**
Cell membrane	0.7588	0.7683	**0.837**

### Prediction of protein subcellular localizations for *F. graminearum*

**Figure 2:**
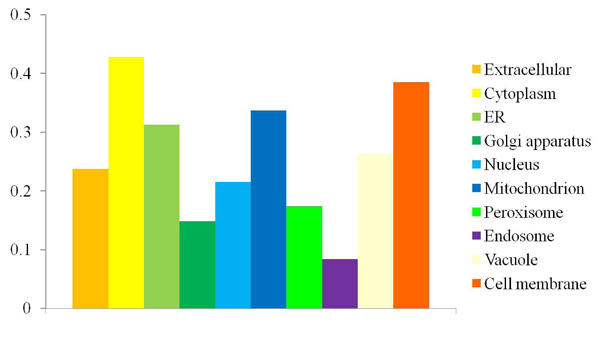
The distribution of proteins among the top 10 subcellular localizations.

After getting the training data set and trained classifier, we aim to predict protein subcellular localizations for *F. graminearum*. First, we predicted *F. graminearum* protein subcellular localizations based on trained classifiers, and assigned 12532 proteins to 10 subcellular localizations. Second, BLAST was employed to predict *F. graminearum* protein subcellular localizations based on protein sequence similarity among *F. graminearum* proteins and fungi proteins with known subcellular localizations so that this can complement SVMs based methods. Moreover, other subcellular localizations with few known proteins were also considered except the 10 localizations used to train classifiers. As a result, 4897 more proteins were assigned to 24 subcellular localizations. Finally, there are 12786 proteins in total that were assigned to at least one of 24 protein subcellular localizations. In summary, our predictions cover about 95.98% (12786 out of 13321 proteins) of *F. graminearum* proteins. Table 4 shows the distributions of *F. graminearum* proteins among 24 cellular compartments. Figure 2 shows the distribution of proteins among the top 10 subcellular localizations with largest populations of proteins. It was found that the largest population of proteins belong to the Cytoplasm, in which most biochemical reactions tack place. We are surprised to find that a large number of proteins are predicted to belong to cell membrane. Although there are possible false positives in the predictions, it is reasonable that *F. graminearum* invades plant host, receives signal, and gets nutrients from host with membrane proteins. The third largest population of proteins are found to belong to Mitochondrion which is also found to be the third largest compartment in *Saccharomyces cerevisiae*.

**Table 4:** Distributions of the predicted subcellular localizations for 12786 *F. graminearum* proteins based on ensemble classifier and BLAST.

Localization	Ensemble classifier	BLAST	Ensemble classifier+BLAST
Extracellular	3105	262	3163
Cytoplasm	4782	2050	5699
ER	4016	520	4166
Golgi apparatus	1773	246	1975
Nucleus	1381	1858	2868
Mitochondrion	4115	952	4484
Peroxisome	2202	154	2315
Endosome	1075	52	1114
Vacuole	3377	262	3505
Cell membrane	5035	346	5130
Bud		11	11
Bud neck		36	36
Bud tip		6	6
Lipid-anchor		61	61
Centromere		23	23
Kinetochore		28	28
Telomere		19	19
cytoskeleton		88	88
Spindle		48	48
Prospore membrane		4	4
Peripheral membrane		280	280
Multi-pass membrane		968	968
Single-pass membrane		229	229
Preautophagosomal structure membrane		4	4

Total	12532	4897	12786

Although the predictions need to be verified in lab and there are possible false positives, we believe that the predicted *F. graminearum* protein subcellular localizations can guide future experiments and provide insights into protein function and pathogenic process underlying *F. graminearum*-host interactions. The prediction results are available at http://csb.shu.edu.cn/fgsub/.

### Validation of predicted *F. graminearum* protein subcellular localizations

In order to validate the predicted protein subcellular locations, we investigated the functions of *F. graminearum* proteins with the assumption that proteins in the same cellular compartment should have similar functions. Right now, there are 4321 proteins that have been annotated in MIPS *Fusarium graminearum* Genome DataBase (FGDB) [32]. To see whether a pair of proteins have similar functions, the functional similarity score proposed in our previous work [33] was adopted here, which is defined as:

(1)

where s(*i*, *j*) is the functional similarity score for a protein pair (*i*, *j*), *f* (*i*) represents the set of functional terms from FGDB for protein *i*, ∩ is the intersection of two sets, U is the union of two sets, and |·| means the number of elements in the set, i.e. cardinality of the set. The higher *s*(*i*, *j*) is, the higher confidence that the protein pair (*i*, *j*) have similar functions.

**Figure 3:**
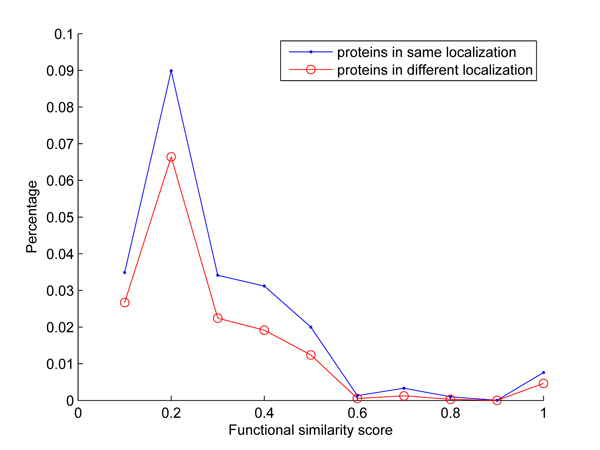
Distributions of functional similarity for protein pairs located to the same subcellular localizations and different subcellular localizations, respectively.

In this work, all possible protein pairs predicted to the same subcellular location were compared against all possible pairs predicted to different subcellular locations. Figure 3 shows the distribution of functional similarity scores for proteins located to same cellular compartment against those located to different cellular compartments, where percentage means the percentage of pairs that have similar functions with similarity score *s*(*i*, *j*). It can be seen from Figure 2 that the proteins located to same organelle more likely have similar functions than those located to different organelles, which validates the reliability of our predicted subcellular locations.

Furthermore, to verify our predicted results, we use the core protein-protein interaction (PPI) data set predicted for *F. graminearum* in our previous work [33]. In order to interact, two proteins must co-occur spatially and temporally, and therefore proteins interacting *in vivo* should be co-located in the same cellular compartment. In literature, Shin et al [34] have used protein-protein interaction data to predict subcellular locations, which proves that proteins in the same compartment are more likely to interact with each other. In the core PPI data set, there are 27102 protein-protein interactions in total. After examining all the pairs belonging to the same subcellular localizations, it is found that 19695 of 27102 pairs are located in same cellular compartments while just 4197 pairs are located in different cellular compartments. The remaining pairs contain proteins that are not predicted to any subcellular locations. As to the 12786 predicted proteins, there are total 81,734,505 pairs, of which 40,067,528 pairs are predicted in same cellular compartments. A p-value less than 10^−15^ was obtained based on the hypergeometric distribution defined as following.

 (2)

Where *N* = 12786 × 12785/2 = 81734505, *n* = 19659 + 4197 = 23892, *M* = 40067528, *m* = 19659. The *p*-value shows a statistical significant difference, which demonstrates that a majority of our predictions are correct.

## Conclusions

In this work, a novel predictor, namely FGsub, is proposed to predict *F. graminearum* protein subcellular localizations from the primary structures in a comprehensive manner. We make four fold contributions to this filed. First, we present a new algorithm to cope with imbalance problem that arises in protein subcellular localization prediction, which can solve imbalance problem and minimize false positive results. Second, we design an ensemble classifier which employs feature selection to further improve prediction accuracy. The 10-fold cross-validations and comparison with other methods demonstrate that our proposed methods are indeed effective for predicting protein subcellular localizations. Third, we use BLAST to complement machine learning based methods, which enlarges our prediction coverage. The two methods complement each other and therefore make the predictions more effective. Last and most important, we predict the subcellular locations of 12786 *F. graminearum* proteins, which provide insights into protein functions and pathogenic mechanisms of this destructive pathogen fungus. Although these predictions are not verified in lab, we believe that the predictions can provide guidelines for future experiments and help to understand this destructive fungus *F. graminearum*.

## Methods

### Data sets

In this work, the annotation of protein subcellular localization for fungi were downloaded from the UniProtKB [30] database release 57.1 and used as training data set for predicting *F. graminearum* protein subcellular localizations. The number of proteins in the data set is 23228, of which 17769 is annotated. After discarding those subcellular location annotations followed ‘By similarity', ‘Potential’ and ‘Probable’, there are 10554 proteins left and were used as reference data set, and these proteins belong to 24 subcellular localizations. Since there are some compartments with few annotated proteins (less than 30), it is not suitable to build classifiers for these compartments due to the small number of samples. Finally, in this paper, 10 subcellular localizations were kept for machine learning based methods, including Extracellular, Cytoplasm, Nucleus, Mitochondria, Endoplasmic reticulum, Golgi apparatus, Peroxisome, Endosome, Vacuole and Cell membrane. Furthermore, proteins located in more than one subcellular compartment were removed, those with less than 50 amino acids in length were removed, and those with ambiguous amino acids (B, X and Z) were also removed from the data set. In addition, CD-HIT program [35] was used to remove the homology bias in the data set with a threshold identity of 40%. Finally, a non-redundant data set of 4592 proteins were obtained and used as training set for machine learning based methods. Table 1 shows the statistics of the ten compartments used in this work.

### Feature extraction and selection

In machine learning, each protein should be represented as a feature vector. In this work, the amino acid triplet (threAA), gapped amino acid composition (GapAA) [13] , N-terminal and C-terminal sequence information are considered here.

For threAA information, each protein vector is generated consisting of frequency of all possible combinations of three amino acids from 20-amino acid alphabet. Therefore, each protein contains 8000 (20^3^) features. For gapped amino acid composition, GapAA *XdY* (*d* ≥ 1) denotes the peptide of length *d* + 2, where amino acids *X* and *Y* are separated by *d* amino acids. Given an upper bound of gapped distance *I*, i.e. 1 ≤ *d* ≤ *l*, each protein can be represented as a vector consists of all possible combinations of GapAA *XdY* (1 ≤ *d* ≤ *l*). Therefore, each protein can be represented as a *l* × 20 × 20-dimensionality vector, where each feature represents the frequency of GapAA appearing in the sequence. For N-terminal and C-terminal features, the first 20 and 40 residues are used for N-terminal composition, and the first 20 and 50 residues are used for C-terminal composition. Furthermore, amino composition (AA), paired amino acids (pAA), one-gapped PAA (Gap1AA), two-gapped PAA (Gap2AA) and three-gapped PAA (Gap3AA) are used as features for N-terminal and C-terminal compositions, respectively. Therefore, each protein is a vector consist of 3240 features for N-terminal and C-terminal compositions respectively.

In addition, to reduce effect of protein sequence length, each feature value is normalized as following:

(3)

where *V_ij_* is the value for feature *j* in vector *i*, *m* is the dimensionality of the vector *i*, where *j* ∊ {1, …, *m*}. The representations of each protein described above have thousands of features, which leads to high computation cost and the noise in the data generally degrade the performance of classifiers. To find out informative features and reduce computation cost, we first utilize *t*-test to rank the features and then employ sequential forward feature selection to select the most informative features starting from the top ranked features by *t*-test. The obtained feature set is used in sequel.

### Re-balancing imbalanced data set

**Figure 4:**
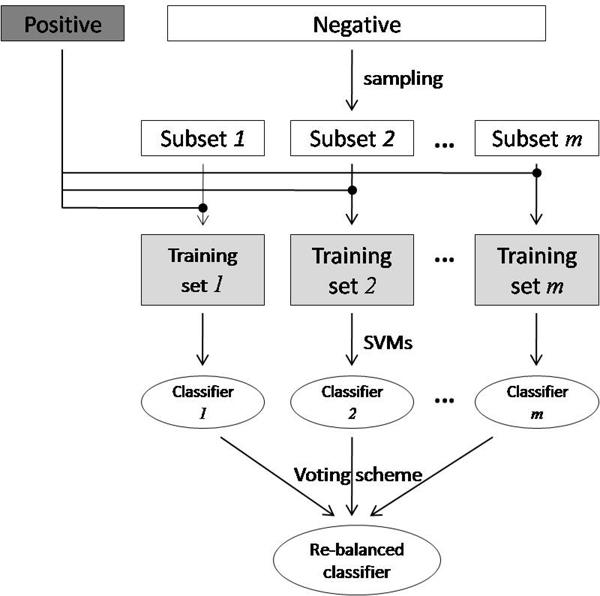
The schematic flowchart of the proposed method for re-balancing the imbalanced data set.

After getting the feature vectors for protein sequences, one classifier can be designed for each localization, and the new protein sequence can be classified into one or more subcellular localizations or none of the ten localizations. However, as described previously, the imbalanced problem will arise in this case. To overcome this problem, a bagging-like re-balanced classifier is presented in this section. Figure 4 presents the schematic flowchart of the proposed method for re-balancing the imbalanced data set. In our work, the number of negative data is always larger than that of positive data, so the negative data set is first under-sampled and divided into *m* subsets, where each subset has similar size as the positive data set. After the sampling procedure, we get *m* training sets, where each training set consists of one subset from the negative data and the positive data, i.e. {positive set, negative subset 1}, ..., {positive set, negative subset *m*}. With the newly generated data sets, we train *m* classifiers with one for each training set. Given a new test example, the prediction results are obtained by fusing the outputs from the *m* classifiers, where the results are combined by a voting scheme.

### Ensemble classifier

**Figure 5:**
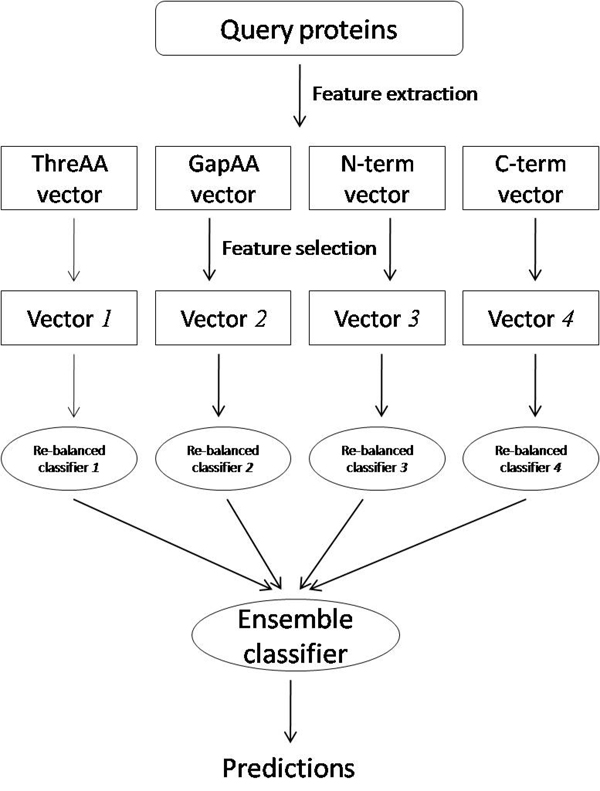
The schematic flowchart of predicting query proteins by ensemble classifier.

With different feature extraction methods described above, each protein is described in a different way. It has been shown that different descriptions for proteins can lead to different results. Generally, there is no guarantee that one single method can always outperform other methods in any cases. On the other hand, these methods may complement each other, and the combination of these methods may lead to better results. In this work, we combine classifiers trained in different feature spaces introduced by different feature extraction methods. Figure 5 shows the schematic flowchart for constructing ensemble classifier. As shown in figure 4, each classifier has inputs with feature descriptions that are different from those to the other classifiers. Consequently, *n* classifiers can be constructed if there are *n* different ways to describe the protein sequences. For a new test example, the combination of outputs from the *n* classifiers is the final decision. In this paper, the weighted majority voting method is adopted here, where the weight for each classifier is the AUC score obtained by each classifier on the training set using 10-fold cross-validation.

### Predicting protein subcellular localization for *F. graminearum*

After getting the ensemble classifiers trained on the fungi data set with localization annotations, these classifiers can be used to predict *F. graminearum* protein subcellular localizations especially for those proteins with low sequence similarity with known proteins. Furthermore, subcellular localization tends to be evolutionarily conserved, and the homologous with localization annotation appears to be a good indicator of the target protein. We therefore use a homology method, in which a BLAST search of a submitted protein is carried out against our database of 10554 proteins with known localization using an E-value cutoff of 1e-10. The localization information of its homologous proteins is then transferred to the target protein.

### Verification of predicted results

To validate the predicted protein subcellular localizations, we investigate the functions of proteins with the assumption that proteins in the same cellular compartment generally have similar functions. *F. graminearum* annotations were downloaded from MIPS FGDB [32], where a small number of genes have been annotated with different functions. Generally, the pair of proteins belonging to the same cellular compartment should have similar functions. Therefore, function enrichment analysis can verify prediction results to some extent. The function enrichment was investigated for the protein pairs predicted to belong to the same compartment or different ones . We compared the proportion of protein pairs sharing at least one function term for proteins predicted to same compartments against those predicted to different ones. Furthermore, to verify our predicted results, we use the core PPI data set of FPPI [33]. It is found that in order to interact two proteins must co-occur spatially and temporally. Therefore, proteins interacting *in vivo* should be generally co-located in the same cellular compartment. In other words, proteins in the same subcellular localization are more likely to interact with each other.

## Competing interests

The authors declare that they have no competing interests.

## Authors' contributions

XMZ conceived the basic idea. CLS conducted the experiments and wrote the manuscript. XMZ, WHT and LNC participated in system design, provided valuable comments, and helped to draft the manuscript.

## References

[B1] Goswami RS Kistler HC Heading for disaster: Fusarium graminearum on cereal crops. Molecular Plant Pathol 2004 5 515 10.1111/j.1364-3703.2004.00252.x 20565626

[B2] Priest FG Campbell I In Brewing Microbiology 2002 3 Springer

[B3] Bennett JW Klich M Mycotoxins. Clinical Microbiology Reviews 2003 16 497 516 12857779 10.1128/CMR.16.3.497-516.2003 PMC164220

[B4] Christina AC The Fusarium graminearum Genome Reveals a Link Between Localized Polymorphism and Pathogen Specialization. Science 2007 317 1400 1402 10.1126/science.1143708 17823352

[B5] Nakai K Horton P PSORT: a program for detecting sorting signals in proteins and predicting their subcellular localization. Trends Biochem Sci 1999 24 34 35 10.1016/S0968-0004(98)01336-X 10087920

[B6] Huang Y Li YD Prediction of protein subcellular locations using fuzzy k-NN method. Bioinformatics 2004 20 21 28 10.1093/bioinformatics/btg366 14693804

[B7] Lee KY Chuang HY Beyer A Sung MK Huh WK Lee B Ideker T Protein networks markedly improve prediction of subcellular localization in multiple eukaryotic species. Nucleic Acids Res 2008 10 1 13 10.1093/nar/gkn619 PMC2582614 18836191

[B8] Nair R Rost B Better prediction of sub-cellular localization by combining evolutionary and structural information. Proteins 2003 53 917 930 10.1002/prot.10507 14635133

[B9] Reinhardt A Hubbard T Using neural networks for prediction of the subcellular location of proteins. Nucleic Acids Res 1998 26 2230 2236 9547285 10.1093/nar/26.9.2230 PMC147531

[B10] Emanuelsson O Nielsen H Brunak S G H Predicting subcellular localization of proteins based on their n-terminal amino acid sequence. Journal of Molecular Biology 2000 300 1005 1016 10.1006/jmbi.2000.3903 10891285

[B11] Chou KC Prediction of protein cellular attributes using pseudo-amino acid composition. Proteins 2001 43 246 255 10.1002/prot.1035 11288174

[B12] Park KJ Kanehisa M Prediction of protein subcellular locations by support vector machines using compositions of amino acids and amino acid pairs. Bioinformatics 2003 19 1656 1663 10.1093/bioinformatics/btg222 12967962

[B13] Chang JM Su ECY Lo A Chiu HS Sung TY Hsu WL PSLDoc: Protein subcellular localization prediction based on gapped-dipeptides and probabilistic latent semantic analysis. Proteins 2008 72 693 710 10.1002/prot.21944 18260102

[B14] Scott MS Thomas DY Hallett MT Prediction subcellular localization via protein motif co-occurrence. Genome Research 2004 14 1957 1966 15466294 10.1101/gr.2650004 PMC524420

[B15] Gardy JL Spencer C Wang K Ester M Tusnady GE Simon I Hua Sj PSORT-B: improving protein subcellular localization prediction for Gramnegative bacteria. Nucleic Acids Res 2003 31 3613 3617 12824378 10.1093/nar/gkg602 PMC169008

[B16] Garga P Sharmaa V Chaudharia P Roy N SubCellProt: Predicting Protein Subcellular Localization Using Machine Learning Approaches. InSilico Biology 2009 9 35 44 19537160

[B17] Hua SJ Sun ZR Support vector machine approach for protein subcellular localization prediction. Bioinformatics 2001 17 721 728 10.1093/bioinformatics/17.8.721 11524373

[B18] Wang J Sung WK Krishnan A Li kB Protein subcellular localization prediction for Gram-negative bacteria using amino acid subalphabets and a combination of multiple support vector machines. BMC Bioinformatics 2005 6 174 16011808 10.1186/1471-2105-6-174 PMC1190155

[B19] Höglund A Donnes P Blum T W AH O K Multiloc: prediction of protein localization using n-terminal targeting sequences, sequence motifs and amino acid compositions. Bioinformatics 2006 22 1158 1165 10.1093/bioinformatics/btl002 16428265

[B20] Tamura T Akutsu T Subcellular location prediction of proteins using support vector machines with alignment of block sequences utilizing amino acid composition. BMC Bioinformatics 2007 8 466 18047679 10.1186/1471-2105-8-466 PMC2220007

[B21] Cedano J Aloy P Perez-Pons JA Querol E Relation between amino acid composition and cellular location of proteins. Journal of Molecular Biology 1997 266 3 594 600 10.1006/jmbi.1996.0804 9067612

[B22] Nakashima H Nishikawa K Discrimination of Intracellular and Extracellular Proteins Using Amino Acid Composition and Residue-pair Frequencies. Journal of Molecular Biology 1994 238 54 61 10.1006/jmbi.1994.1267 8145256

[B23] Bhasin M Raghava GPS ESLpred: SVM-based method for subcellular localization of eukaryotic proteins using dipeptide composition and PSI-BLAST. Nucl. Acids Res 2004 32 suppl 2 W414 419 10.1093/nar/gkh350 PMC441488 15215421

[B24] Chen H Huang N Sun Z SubLoc: a server/client suite for protein subcellular location based on SOAP. Bioinformatics 2006 22 3 376 377 10.1093/bioinformatics/bti822 16339283

[B25] Horton P Park K Obayashi T Fujita N Harada H Adams-Collier C Nakai K WoLF PSORT: Protein Localization Predictor. Nucleic Acids Research 2007 [Doi:10.1093/nar/gkm259] 10.1093/nar/gkm259 PMC1933216 17517783

[B26] Cai Y Chou K Predicting subcellular localization of proteins in a hybridization space. Bioinformatics 2004 20 1151 1156 10.1093/bioinformatics/bth054 14764553

[B27] Pierleoni A Martelli PL Fariselli P Casadio R BaCelLo: a balanced subcellular localization predictor. Bioinformatics 2006 22 14 e408 416 10.1093/bioinformatics/btl222 16873501

[B28] Liu J Kang S Tang C Ellis LB Li T Meta-prediction of protein subcellular localization with reduced voting. Nucleic Acids Res 2007 35 15 e96 17670799 10.1093/nar/gkm562 PMC1976432

[B29] Zhao XM Chen LN Aihara K Protein classification with imbalanced data. Proteins 2008 4 1125 1132 10.1002/prot.21870 18076026

[B30] Consortium TU The Universal Protein Resource (UniProt). Nucleic Acids Res 2009 37 D169 D174 18836194 10.1093/nar/gkn664 PMC2686606

[B31] Chang CC Lin CJ LIBSVM: a library for support vector machines 2001. Software available at http://www.csie.ntu.edu.tw/~cjlin/libsvm

[B32] Guldener U Mannhaupt G Munsterkotter M Haase D Oesterheld M Stumpflen V Mewes HW Adam G FGDB: a comprehensive fungal genome resource on the plant pathogen Fusarium graminearum. Nucleic Acids Res 2006 34 D456 D458 16381910 10.1093/nar/gkj026 PMC1347389

[B33] Zhao XM Zhang XW Tang WH Chen LN FPPI:Fusarium graminearum Protein-Protein Interaction Database. Journal of Proteome Research 2009 8 4714 4721 10.1021/pr900415b 19673500

[B34] Shin CJ Wong S Davis MJ Ragan MA Protein-protein interaction as a predictor of subcellular location. BMC Systems Biology 2009 3 28 19243629 10.1186/1752-0509-3-28 PMC2663780

[B35] Li WZ Godzik A Cd-hit: a fast program for clustering and comparing large sets of protein or nucleotide sequences. Bioinformatics 2006 22 1658 1659 10.1093/bioinformatics/btl158 16731699

